# EMI characteristics analysis and suppression technique of magnetic near-field coupling in power delivery adapter

**DOI:** 10.1038/s41598-022-11977-0

**Published:** 2022-05-11

**Authors:** Qingbin Chen, Dandan Zhang, Wei Chen

**Affiliations:** College of Electrical Engineering and Automation, Fuzhou University, Fuzhou, 350108 China

**Keywords:** Electrical and electronic engineering, Applied physics

## Abstract

With the continuous improvement of the power density of the power delivery (PD) adapter, switching frequency increases and volume decreases. The EMI issues caused by magnetic near-field coupling effects have become bottlenecks in the PD adapter’s EMI suppression. In this paper, the coupling characteristics of the magnetic field are deeply analyzed, and the equivalent circuit considering near-field coupling effects is derived. According to the electromagnetic field theory, the mathematical models of near-field coupling between transformer as well as power PCB circuits and the input plug are established. Based on this, the influence of the structure parameters of the input plug is studied. Finally, a new input plug loop structure is proposed, which can reduce the near-field coupling effects between the transformer as well as power PCB circuits and input plug. The experimental results verify the theoretical analysis to be correct and effective.

## Introduction

With the continued increasing power density of the power delivery (PD) adapter, the distance among devices is getting closer and closer. The electromagnetic coupling between two components is becoming enormous. Furthermore, electromagnetic interference (EMI) caused by near-field coupling effects has become a bottleneck in PD adapter’s design^1–3^. The near-field coupling effects are hard to understand and deal with.

As for the influence of magnetic near-field coupling on EMI, research results mainly focus on the effect of near-field coupling between magnetic power components and EMI filter^4–8^. In Ref.^6^, the leakage magnetic field distribution of power factor correction (PFC) choke and the sensitivity of common-mode (CM) choke in a PFC converter are analyzed. It reveals that the insertion loss of the EMI filter may be deteriorated by the magnetic field coupling between PFC and CM chokes. In Ref.^7^, the magnetic field distribution of planar transformer and the influence on CM choke coil in power supply have been analyzed. Moreover, the layout, shielding, and CM choke design method are proposed to reduce the effects of magnetic near-field coupling to suppress EMI noise.

Besides, the characteristic of the EMI filter can also be affected by magnetic near-field coupling between components inside EMI filter^8–11^. In Ref.^8^, near-field coupling between two shunt capacitors in the EMI filter is analyzed, and a negative mutual coupling method is proposed to offset the equivalent series inductance (ESL) of capacitors. In Ref.^9^, the electromagnetic coupling of a CLC EMI filter is analyzed. A method is proposed to balance the parasitic inductive and capacitive coupling between two capacitors. It can optimize the performance of the EMI filter. In Ref.^10^, magnetic field coupling in a two-stage EMI filter is analyzed. A series inductor integration method using near-field coupling effects is proposed to improve the low-frequency performance of the DM EMI filter.

However, the input plug close to the high-frequency power circuits is also a possible noise propagation path due to near-field coupling effects, especially in high power density applications^12–14^. A double-wire parallel wound CM choke in the PD adapter is usually adopted to reduce near-field coupling effects between power magnetic components and CM choke. In this case, near-field coupling paths in the compact PD adapter are pretty different from traditional ones.

Therefore, this paper analyzes near-field coupling characteristics in a compact PD adapter and studies the influence of near-field coupling on DM conducted EMI noise. Magnetic field coupling between transformer as well as PCB power current loops and input plug loop are intensely studied. The mathematical coupling models are built, then a near-field coupling optimization method is proposed. Finally, experiments verify the theory analysis to be correct and flexible.

## Near-field coupling analysis of PD adapter

### Magnetic field coupling between transformer and CM choke

**Figure 1:**
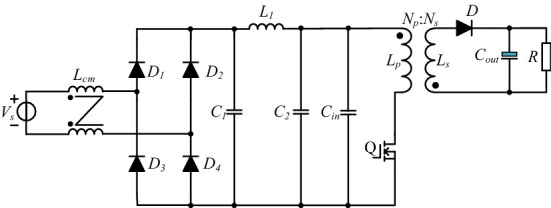
Topology of PD adapter.

The topology of the PD adapter is shown in Fig. 1, including CM choke, rectifier bridge, π type filter, and flyback converter.

**Figure 2:**
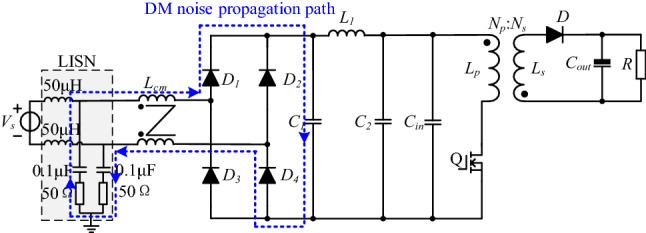
Propagation path of DM noise coupled to the CM choke.

Near-field coupling between transformer and CM choke can be analyzed by the electromagnetic theory, in which the transformer is usually seen as an interference component. In contrast, CM choke is deemed as a sensitive component. The propagation path of DM noise coupled to the CM choke is shown in Fig. 2.

The leakage magnetic field generated from the transformer and coupling to CM choke can be divided into three directions (x, y, and z-direction).

**Figure 3:**
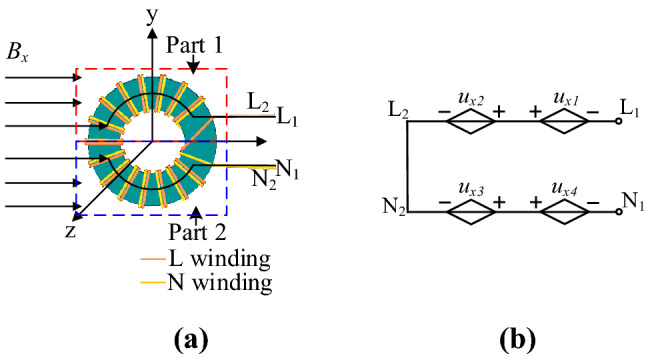
CM choke subjected to the magnetic field in x-direction: (**a**) schematic diagram; (**b**) equivalent circuit diagram.

Figure 3a is a schematic diagram of a double-wire parallel wound CM choke used in the PD adapter under the x-direction magnetic field. The magnetic field chain at the x-direction passes through all the coils. According to Faraday’s law, the voltages induced in two windings are shown in Fig. 3b. Where *u*_*x*1_ and *u*_*x*2_ are the induced voltage generated in part 1 and part 2 of the live wire, *u*_*x*3_ and *u*_*x*4_ are the induced voltage generated in part 1 and part 2 of the naught wire, respectively. Both windings have a symmetrical structure and are tightly wound together. Therefore, the total induced voltages are offset. The CM choke is insensitive to the magnetic field along the x-axis.

**Figure 4:**
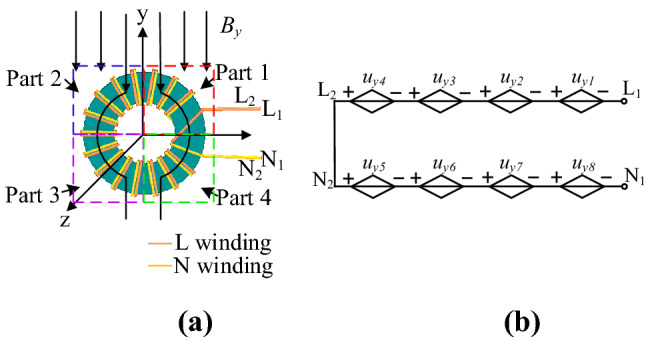
CM choke subjected to the magnetic field in y-direction: (**a**) schematic diagram; (**b**) equivalent circuit diagram.

Figure 4a is a schematic diagram of the CM choke under the y-direction magnetic field. As shown in Fig. 4b, the total induced voltages are offset according to Faraday’s law, where *u*_*y*1_–*u*_*y*4_ are the induced voltage generated in four parts of the live wire, *u*_*y*5_–*u*_*y*8_ are the induced voltage generated in four parts of the naught wire, respectively.

**Figure 5:**
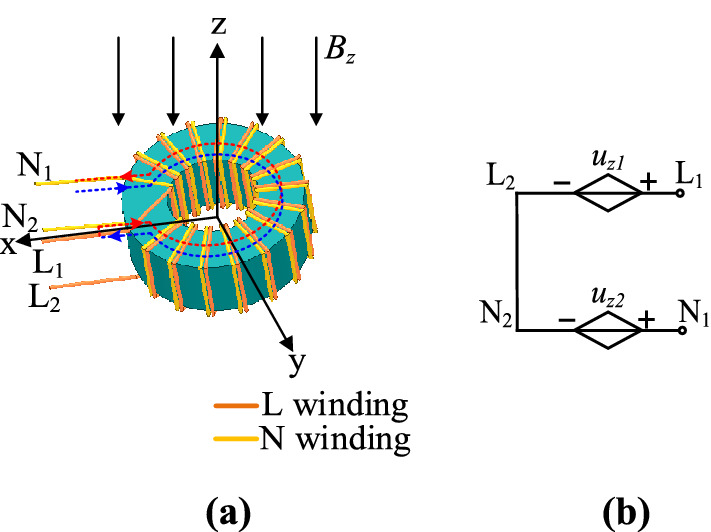
CM choke subjected to the magnetic field in z-direction: (**a**) schematic diagram; (**b**) equivalent circuit diagram.

Figure 5a is a schematic diagram of the CM choke under the z-direction magnetic field. The CM choke under the z-direction magnetic field is equivalent to a loop with two-turn winding perpendicular to the z-direction. The induced voltage of each turn coil generated under the z-direction magnetic field is offset in Fig. 5b, where *u*_*z*1_ and *u*_*z*2_ are the induced voltage generated in live wire and naught wire, respectively.

Above all, the double-wire parallel wound CM choke has high immunity to external magnetic field interference. Therefore, near-field coupling between transformer and CM choke is usually at a low level and can be neglected. Similarly, the magnetic field coupling between DM choke and CM choke can also be analyzed by the same method. It can usually be neglected too.

### Analysis of complete coupling model

**Figure 6:**
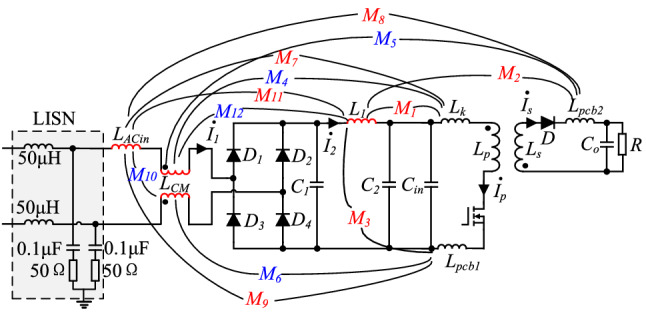
Complete near-field coupling model of the PD adapter.

As to near-field coupling in PD adapter, not only magnetic components in the circuits need to be considered, but also input plug and high-frequency power PCB circuits. The complete near-field coupling model of the PD adapter is shown in Fig. 6. Where *L*_*Acin*_ is the parasitic inductance of the input plug, *L*_*CM*_ is the inductance of CM choke, *L*_*1*_ is the inductance of DM choke, *L*_*pcb*1_ and *L*_*pcb*2_ are the parasitic inductance of the primary and secondary power PCB loops. Near-field coupling between other devices and these three devices is taken into consideration. *M*_1_–*M*_12_ are the mutual inductance between every two components or loops, respectively.

**Figure 7:**
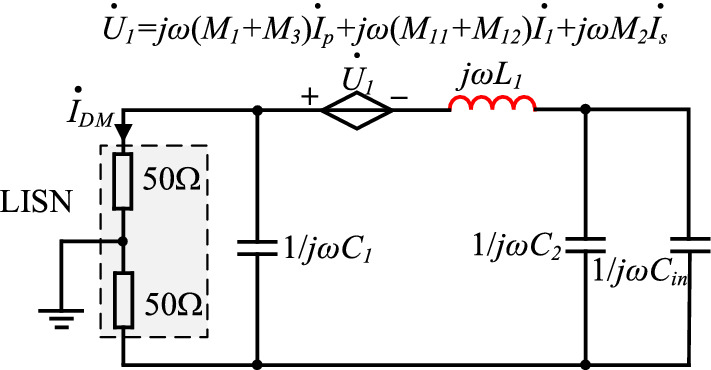
DM noise equivalent circuit diagram considering the effects of near-field coupling on DM choke.

Near-field coupling between magnetic components in the PD adapter and the primary and secondary power PCB loops and DM choke *L*_1_ can be simplified into a corresponding current-controlled voltage source, and its equivalent circuit is shown in Fig. 7.

Since there are two X capacitors *C*_1_ and *C*_2_ at both ends of the DM choke, the noise coupled to *L*_1_ can be bypassed by *C*_1_.

**Figure 8:**
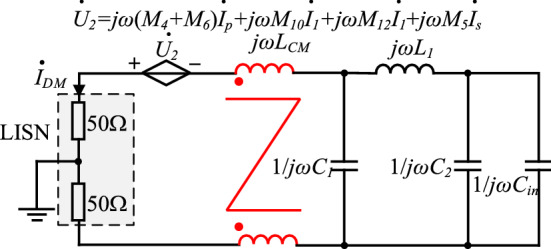
DM noise equivalent circuit diagram considering the effects of near-field coupling on CM choke.

The equivalent circuit diagram of near-field coupling between CM choke *L*_*CM*_ and magnetic components in the PD adapter, as well as the primary and secondary power PCB loops, is shown in Fig. 8.

The two-wire parallel wound CM choke *L*_*CM*_ has high immunity to the interference of near-field coupling, so near-field coupling between CM choke and other components, or loops can be neglected.

**Figure 9:**
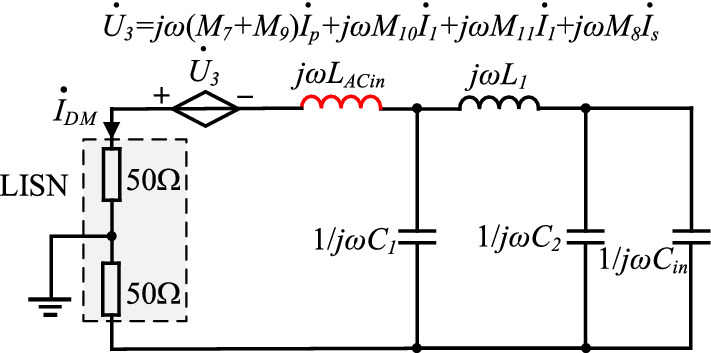
DM noise equivalent circuit diagram considering near-field coupling.

Near-field coupling between the input plug and magnetic components in the PD adapter, as well as the primary and secondary power PCB circuits, can be simplified into a corresponding current-controlled voltage source, and its equivalent circuit is shown in Fig. 9.

From Fig. 9, the noise current on parasitic inductor *L*_*ACin*_ of the input plug can directly flow into LISN and form DM noise.

Above all, near-field coupling between high-frequency power PCB circuits and other components of the PD adapter and the input plug will be the critical factor, and it may affect the EMI performance of the PD adapter.

## Near-field coupling model of PD adapter

### The model of magnetic field coupling between transformer and input plug loop

Near-field coupling between transformer leakage inductance and the input plug loop can be expressed by mutual inductance *M*_*trans*_, calculated by Eq. (1).

1 $$M_{trans} = \frac{\Psi }{I}.$$

**Figure 10:**
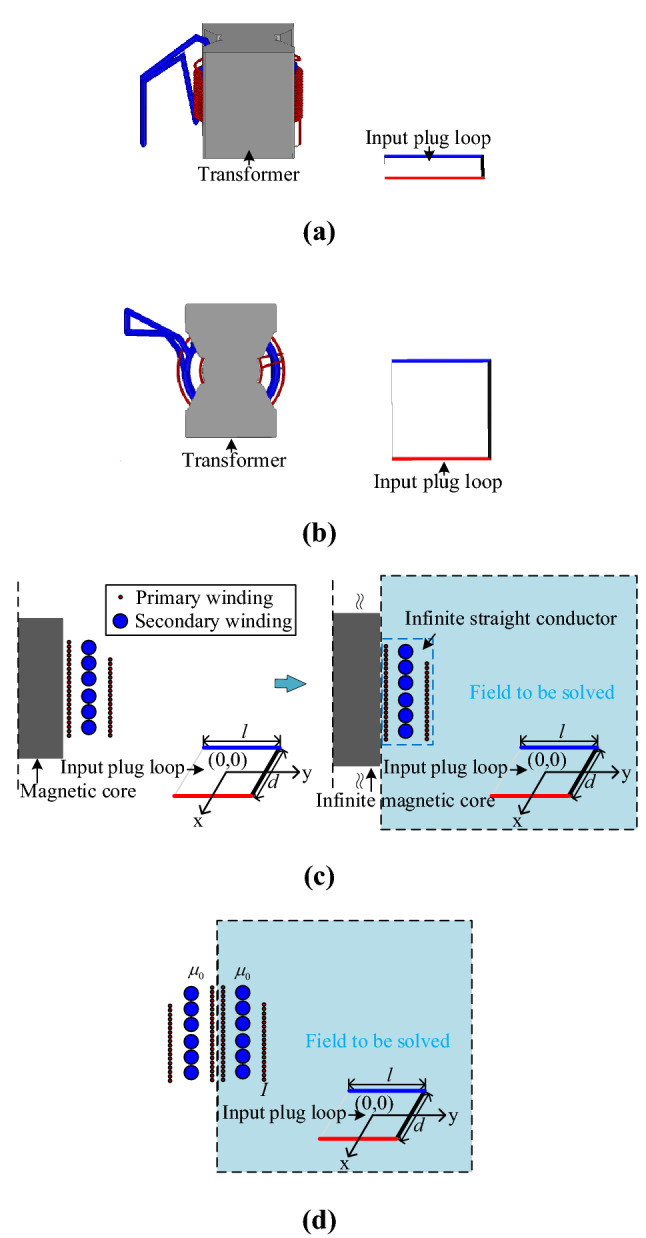
Derivation process of transformer model: (**a**) coupling between transformer and plug; (**b**) xoy plane view of transformer and plug; (**c**) ideal Equivalent of; (**d**) coupling calculation model.

This paper uses the mirror-image method to calculate mutual inductance between the transformer and the input plug loop. The derivation process of the transformer model is shown in Fig. 10, in which the transformer windings are regarded as infinitely long and straight conductors, and the PQ core is regarded as an infinitely sizeable magnetic conduction plane. According to the electromagnetic field “uniqueness” theorem, mirror currents are applied to replace the magnetized current dispersed on the boundary surface. The medium in the field where mirror currents are located is replaced by the medium in the area to be solved. In which, *l* and *d* are the input plug loop’s length and width, respectively.

**Figure 11:**
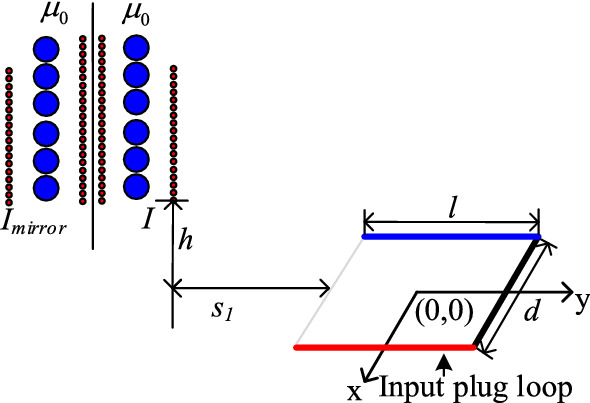
Schematic diagram of magnetic field solved by image method.

Then, the flux density ***B*** of the position where the input plug loop is located can be solved by Ampere’s law, as shown in Fig. 11.

**Figure 12:**
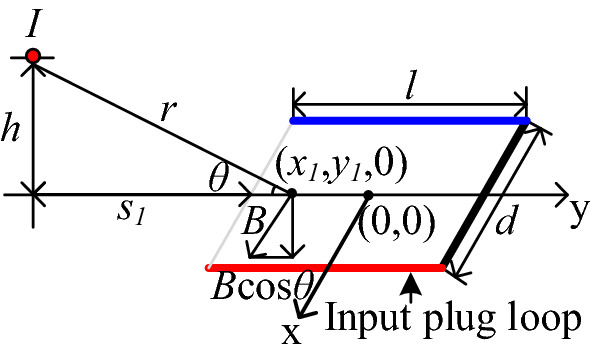
Mutual inductance between each transformer winding and plug loop.

As shown in Fig. 12, the Cartesian coordinate system is adopted where the current *I*_*mirror*_ can be calculated by Eq. (2)^15^.

2 $$I_{mirror} = \frac{{\mu_{1} - \mu_{0} }}{{\mu_{1} + \mu_{0} }}I = I.$$

The flux density produced by each winding turn can be calculated by Eq. (3).

3 $$B = \frac{{\mu_{0} I}}{2\pi r},$$

where, $$r = \sqrt {(b + y_{1} + s_{1} )^{2} + h^{2} }$$.

The flux linkage generated by each winding turn passing through the input plug loop is:

4 $$\begin{aligned} \Psi = & \int_{ - a}^{a} {\int_{ - b}^{b} {B \cdot \cos \theta dy_{1} } } dx_{1} \\ = & \int_{ - a}^{a} {\int_{ - b}^{b} {\frac{{\mu_{0} I \cdot \left( {b + y_{1} + s_{1} } \right)}}{{2\pi \cdot \left( {(b + y_{1} + s_{1} )^{2} + h^{2} } \right)}}dy_{1} } } dx_{1} . \\ \end{aligned}$$

The mutual inductance between the transformer and the input plug loop is equal to the magnitude of magnetic flux linked in the input plug loop when the unit current of the primary windings. In this situation, the induced current of secondary windings is $\frac{{N_{p} }}{{N_{s} }}I_{p}$. Hence, *M*_*trans*_ can be expressed by Eq. (5).

5 $$\begin{aligned} M_{trans} = & \sum\limits_{n = 1}^{{2N_{p} }} {\int_{ - a}^{a} {\int_{ - b}^{b} {\frac{{\mu_{0} \cdot \left( {b + y_{1} + s_{1n} } \right)}}{{2\pi \cdot \left( {(b + y_{1} + s_{1n} )^{2} + h_{n}^{2} } \right)}}dy_{1} } } dx_{1} } \\ & + \sum\limits_{{n = 2N_{p} + 1}}^{{2N_{p} + 2N_{s} }} {\int_{ - a}^{a} {\int_{ - b}^{b} {\frac{{\mu_{0} \frac{{N_{p} }}{{N_{s} }} \cdot \left( {b + y_{1} + s_{1n} } \right)}}{{2\pi \cdot \left( {(b + y_{1} + s_{1n} )^{2} + h_{n}^{2} } \right)}}dy_{1} } } dx_{1} .} \\ \end{aligned}$$

**Figure 13:**
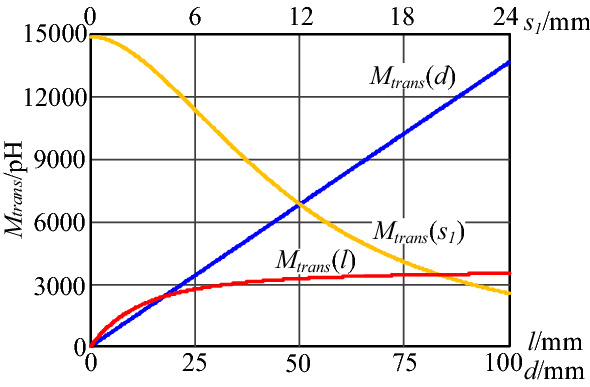
Calculation results of mutual inductance *M*_*trans*_ varies with plug structure parameters.

Keeping two of three parameters the length *l* of the input plug, the width *d* between plug pins, and the distance *s*_1_ between the transformer and plug fixed. The variation of mutual inductance *M*_*trans*_ with *l*,* d* and *s*_1_ is shown in the figure shown in Fig. 13. Where *l* is 80 mm, *d* is 25 mm, *s*_1_ is 16.65 mm.

As shown in Fig. 13, the mutual inductance *M*_*trans*_ increases as *l* and *d* increase. At the same time, *M*_*trans*_ decreases with the rise of distance *s*_1_.

**Figure 14:**
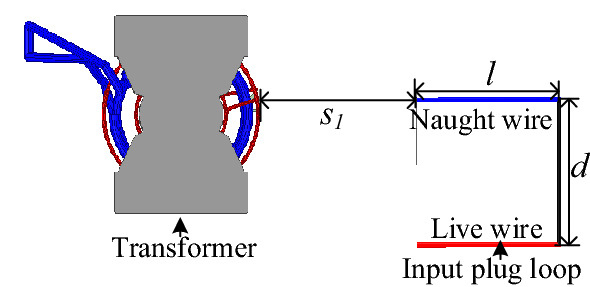
3D FEA simulation model of transformer leakage inductance and input plug loop (xoy plane view).

However, the established equivalent model of the near-field coupling between the transformer and the input plug loop is different from the actual structure. The magnetic core and winding turns cannot be infinite, and some other components are close. Hence, the actual mutual inductance *M*_*trans*_ between transformer and input plug loop needs to be measured or simulated. The finite element analysis (FEA) simulation model is shown in Fig. 14.

**Figure 15:**
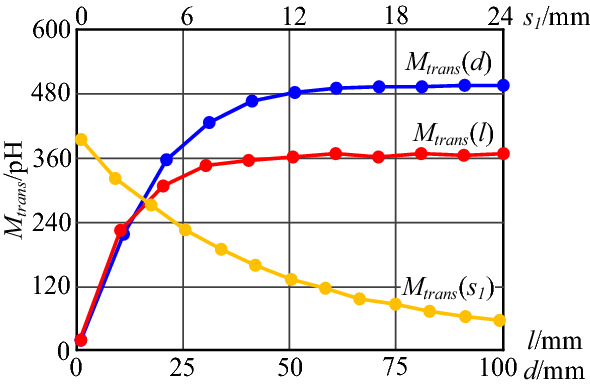
Simulation results of mutual inductance *M*_*trans*_ varies with plug structure parameters.

The mutual inductance *M*_*trans*_ varying with the plug structure parameters can be obtained through simulation analysis, as shown in Fig. 15.

As shown in Fig. 15, the variation trends of *M*_*trans*_ obtained by simulation with *l* and *s*_1_ is the same as model calculation results. However, the variation trend of *M*_*trans*_ obtained by simulation with *d* is somewhat different from the model calculation result. The model calculation result have shown that *M*_*trans*_ increases linearly with the distance *d* between plug pins. Actually, the transformer windings are not infinite, and the leakage flux generated by the transformer decreases with the distance from the transformer. Therefore, the mutual inductance between the transformer and the input plug loop will not increase infinitely with *d*, but will tend to a constant value. For the above reasons, the values of simulation and calculation have some differences too.

### The model of magnetic field coupling between high-frequency power PCB loops and input plug loop

For the magnetic field coupling between high-frequency power PCB loops and input plug loop, the mutual inductance *M*_*loop*_ can also be used to represent the influence of near-field coupling effects. It can be seen from Eq. (6) that the mutual inductance between two loops is related to the number of turns, shapes, and distance of two loops.

6 $$M = \frac{{N_{1} N_{2} \mu_{0} }}{4\pi }\oint_{{l_{2} }} {\oint_{{l_{1} }} {\frac{{dl_{1} dl_{2} }}{R}} } .$$

**Figure 16:**
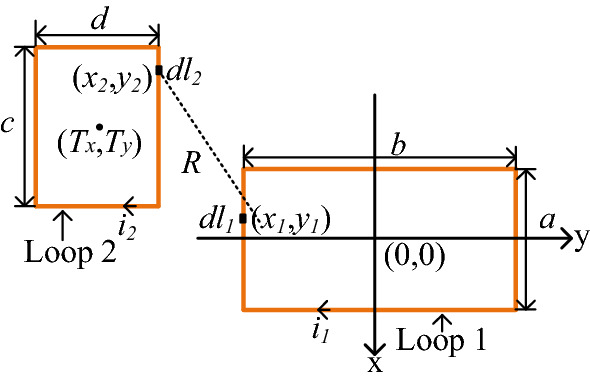
Mutual inductance between two rectangular loops.

The mutual inductance of two rectangular loops in the same plane can be calculated using the Neelman equation. The Cartesian coordinate system is established, as shown in Fig. 16. The center of Loop 1 is placed at the origin of coordinates. The width and length of Loop 1 are *a* and *b*, respectively, while the width and length of Loop 2 are *c* and *d*, respectively. The coordinate of the center point of Loop 2 is (*T*_*x*_, *T*_*y*_).

Coordinate transformation is carried out for formula (6) to calculate the mutual inductance between two loops in the same plane (7).

7 $$\begin{aligned} M_{loop} = & \frac{{\mu_{0} }}{4\pi }\oint_{{l_{2} }} {\oint_{{l_{1} }} {\frac{{(dx_{1} ,dy_{1} ) \cdot (dx_{2} ,dy_{2} )}}{R}} } \\ = & \frac{{\mu_{0} }}{4\pi }\oint_{{l_{2} }} {\oint_{{l_{1} }} {\frac{{dx_{1} dx_{2} + dy_{1} dy_{2} }}{R},} } \\ \end{aligned}$$

where

8 $$x_{2} = T_{x} + x^{\prime},$$

9 $$y_{2} = T_{y} + y^{\prime},$$

10 $$R = \sqrt {\left[ {x_{1} - (T_{x} + x^{\prime})} \right]^{2} + \left[ {y_{1} - (T_{y} + y^{\prime})} \right]^{2} } .$$

According to Eqs. (7)–(10), the mutual inductance *M*_*loop*_ of two rectangular loops is as follows:

11 $$\begin{aligned} M_{loop} = & \frac{{\mu_{0} }}{4\pi } \cdot \bigg(\int_{{ - \frac{a}{2}}}^{\frac{a}{2}} {dx_{1} \int_{{ - \frac{c}{2}}}^{\frac{c}{2}} {\frac{{dx^{\prime}}}{{\sqrt {(x_{1} - (T_{x} + x^{\prime}))^{2} + (b - (T_{y} + d)} )^{2} }}} } \\ & + \int_{{ - \frac{a}{2}}}^{\frac{a}{2}} {dx_{1} \int_{\frac{c}{2}}^{{ - \frac{c}{2}}} {\frac{{dx^{\prime}}}{{\sqrt {(x_{1} - (T_{x} + x^{\prime}))^{2} + (b - (T_{y} - d)} )^{2} }}} } \\ & + \int_{\frac{a}{2}}^{{ - \frac{a}{2}}} {dx_{1} \int_{{ - \frac{c}{2}}}^{\frac{c}{2}} {\frac{{dx^{\prime}}}{{\sqrt {(x_{1} - (T_{x} + x^{\prime}))^{2} + ( - b - (T_{y} + d)} )^{2} }}} } \\ & + \int_{\frac{a}{2}}^{{ - \frac{a}{2}}} {dx_{1} \int_{\frac{c}{2}}^{{ - \frac{c}{2}}} {\frac{{dx^{\prime}}}{{\sqrt {(x_{1} - (T_{x} + x^{\prime}))^{2} + ( - b - (T_{y} - d)} )^{2} }}} } \\ & + \int_{{ - \frac{b}{2}}}^{\frac{b}{2}} {dy_{1} \int_{{ - \frac{d}{2}}}^{\frac{d}{2}} {\frac{{dy^{\prime}}}{{\sqrt {(a - (T_{x} + c))^{2} + (y_{1} - (T_{y} + y^{\prime})} )^{2} }}} } \\ & + \int_{{ - \frac{b}{2}}}^{\frac{b}{2}} {dy_{1} \int_{\frac{d}{2}}^{{ - \frac{d}{2}}} {\frac{{dy^{\prime}}}{{\sqrt {(a - (T_{x} - c))^{2} + (y_{1} - (T_{y} + y^{\prime})} )^{2} }}} } \\ & + \int_{\frac{b}{2}}^{{ - \frac{b}{2}}} {dy_{1} \int_{{ - \frac{d}{2}}}^{\frac{d}{2}} {\frac{{dy^{\prime}}}{{\sqrt {( - a - (T_{x} + c))^{2} + (y_{1} - (T_{y} + y^{\prime})} )^{2} }}} } \\ & + \int_{\frac{b}{2}}^{{ - \frac{b}{2}}} {dy_{1} \int_{\frac{d}{2}}^{{ - \frac{d}{2}}} {\frac{{dy^{\prime}}}{{\sqrt {( - a - (T_{x} - c))^{2} + (y_{1} - (T_{y} + y^{\prime})} \bigg)^{2} }}).} } \\ \end{aligned}$$

**Figure 17:**
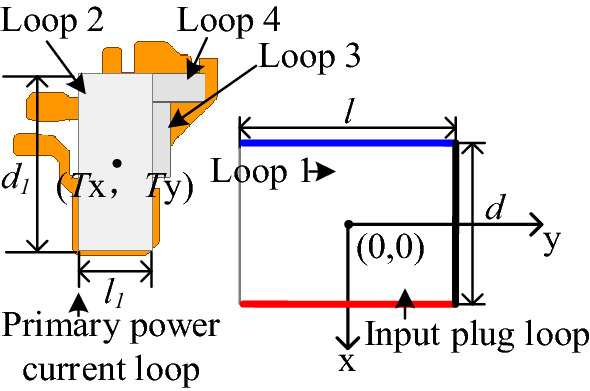
Mutual inductance between primary power PCB loop and plug loop.

Theoretically, a PCB loop with an arbitrary shape can be equivalent to a limited number of rectangular loops. For the convenience of calculation, several rectangle loops with key influences are usually taken to approximate the primary power PCB loop with an equivalent irregular shape. As shown in Fig. 17, the input plug loop is equal to rectangular Loop 1, and the primary power PCB loop is equivalent to three rectangular loops: Loop 2, Loop 3, and Loop 4. The equivalent three rectangular loops have the same current direction as the primary power PCB loop, so the mutual inductance between the primary power PCB loop and input plug loop can be equivalent to the sum of the mutual inductance between Loop 1 and Loop 2, 3, and 4. The total equivalent mutual inductance *M*_*pri*_ is calculated as:

12 $$M_{pri} = M_{loop2} + M_{loop3} + M_{loop4} .$$

PCB traces have a specific width in actual applications, and the current does not flow at the centerline of the PCB traces. They are different from the above theoretical assumptions. Meanwhile, if two loops are not in the same plane, the calculation method is different. The loop needs to be projected to xoy, xoz, and yoz planes.

**Figure 18:**
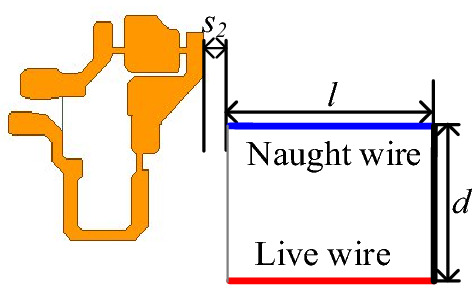
3D FEA simulation model of primary power PCB loop and plug loop (xoy plane view).

The simulation was carried out to explore the influencing factors of mutual inductance between the PD adapter’s power current loops and the input plug loop. Moreover, the influences of different plug structure parameters on near-field coupling are analyzed. The FEA simulation model is shown in Fig. 18.

**Figure 19:**
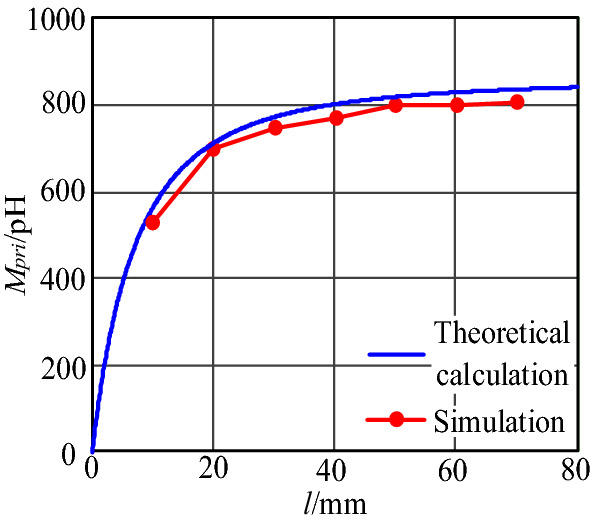
Mutual inductance *M* between two loops varies with *l*.

**Figure 20:**
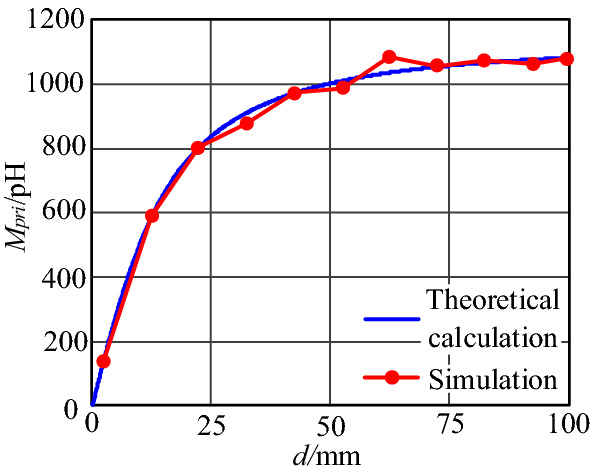
Mutual inductance *M* between two loops varies with *d*.

**Figure 21:**
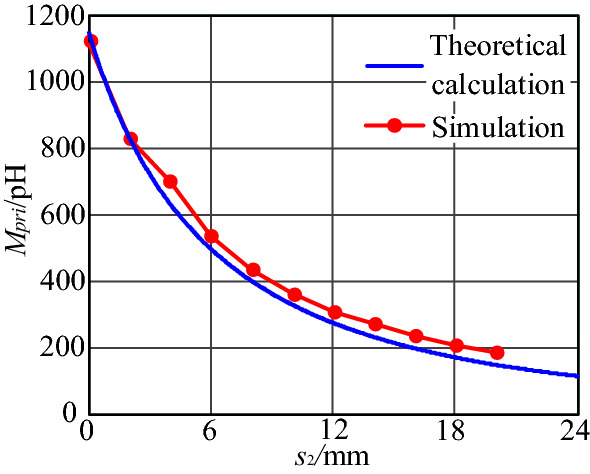
Mutual inductance *M* between two loops varies with *s*_2_.

By keeping two parameters of length *l*, width *d* between plug pins and loop spacings *s*_*2*_ unchanged, the mutual inductance between the primary power PCB loop and input plug loop under different lengths *l*, widths *d*, and distance *s*_2_ can be obtained through simulation. Where *l* is 80 mm, *d* is 25 mm, *s*_2_ is 1.94 mm. The trends of mutual inductance *M*_*pri*_ between the two loops with *d*, *s*_2_, and *l* are plotted in Figs. 19, 20 and 21, respectively, and compared with the results calculated according to the theoretical model.

It can be seen from Fig. 19 that the mutual inductance *M*_*pri*_ between the primary power PCB loop and the input plug loop increases with plug length *l*, and the change rate of *M*_*pri*_ decreases with *l*. When *l* reaches 50 mm, the increment of *M*_*pri*_ with *l* is not apparent. The simulation results are consistent with the theoretical calculation results. Therefore, in this near-field coupling model, the effective length influenced by near-field coupling is 50 mm.

It can be seen from Fig. 20 that with the increase of width *d* between plug pins, *M*_*pri*_ increases, and when *d* is in the range of 0–25 mm, *M*_*pri*_ increases linearly. When *d* reaches 50 mm, it continues to grow, but the increment of *M*_*pri*_ is not apparent. It is consistent with the theoretical calculation results. Therefore, the effective width influenced by near-field coupling is 50 mm.

As shown in Fig. 21, the mutual inductance *M*_*pri*_ decreases with the increase of the distance *s*_2_, and when the distance *s*_2_ increases above 20 mm, the decreasing trend of *M*_*pri*_ of two loops slows down. It is consistent with the theoretical calculation results. Therefore, the effective distance influenced by near-field coupling is 20 mm.

Similarly, magnetic field coupling between the secondary power PCB loop and the input plug loop can be theoretically calculated and simulated by the same method. Then, the total mutual inductance *M*_*total*_ of the PD adapter to the input plug loop is equal to the sum of the mutual inductance of the transformer and the primary and secondary PCB loops to the input plug loop.

## Near-field coupling optimization method

According to the above analysis, it can be known that the effects of near-field coupling on the input plug loop can be suppressed by increasing the distance between the input plug loop and transformer as well as power PCB loops. However, considering the volume of the PD adapter, the application of this method is limited.

On the other hand, the mutual inductance *M*_*total*_ can be reduced by reducing plug length *l* and width *d* between plug pins. However, there are specific requirements for the charger plug length *l* and width *d* in practical application.

**Figure 22:**
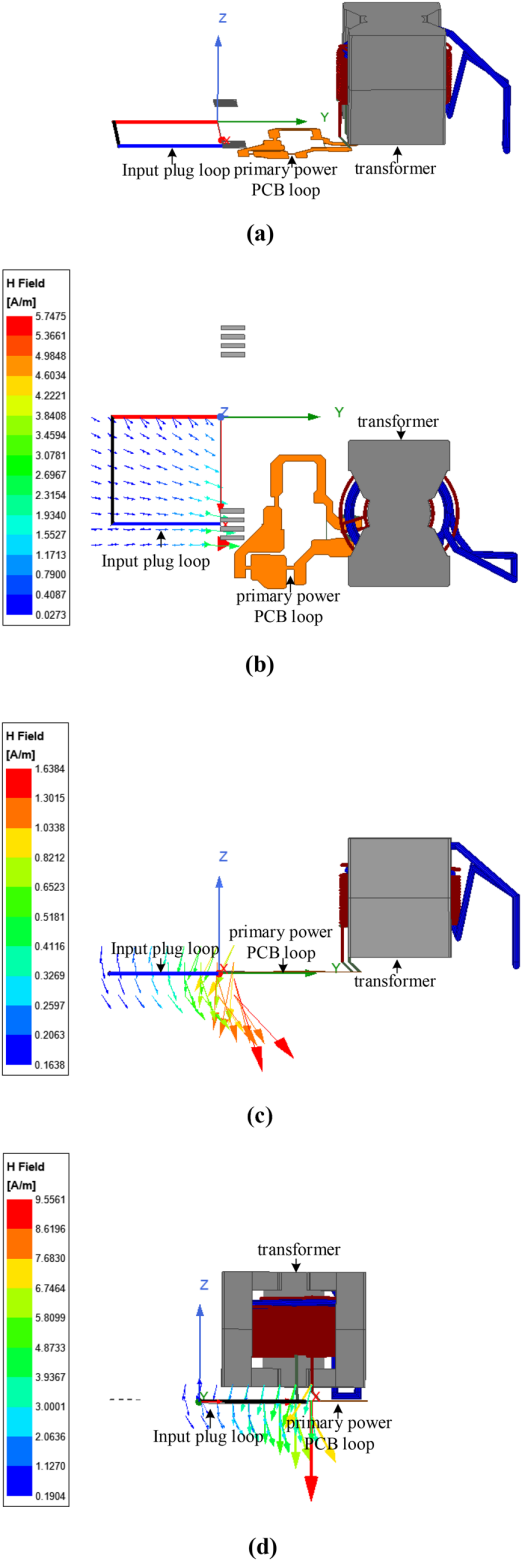
Distribution of magnetic field generated by primary power PCB loop and transformer: (**a**) schematic diagram of magnetic field coupling simulation; (**b**) magnetic field distribution from z-direction; (**c**) magnetic field distribution from x-direction; (**d**) magnetic field distribution from y-direction.

Besides, the *M*_*total*_ is related to the shape of the input plug loop. In this situation, the magnetic field distribution needs to be analyzed firstly. The flux lines generated by the primary power PCB loop and transformer can be obtained through simulation, as shown in Fig. 22. From Fig. 22, most of the flux lines pass through the plug loop vertically in the original structure. If the input plug is rotated until the magnetic force lines are approximately parallel to the plug loop, the influence of near-field coupling on the input plug loop will be significantly reduced.

**Figure 23:**
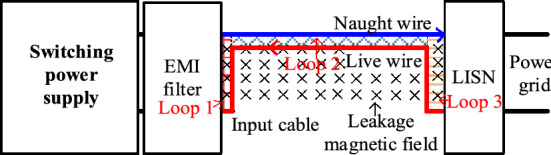
Schematic diagram of the projection of the proposed plug loop structure.

Therefore, considering the distribution of the magnetic field generated by the transformer and the primary power PCB loop and the influence of the effective length on the near-field coupling, a new input plug structure is proposed. The horizontal distance between two plug pins within the effective length range can be reduced, and the vertical distance between two plug pins is increased to satisfy the safety requirements. If necessary, the live and naught wires can be separated at the end, as shown in Fig. 23.

By dividing the projection of the designed structure into several equivalent rectangles, the influence of near-field coupling on the input plug loop can be calculated by a complete near-field coupling model, and total mutual inductance is equal to the sum of mutual inductance. Because the distance between plug Loop 3 and adapter is far, the influence of near-field coupling on plug Loop 3 usually can be neglected. Therefore, the mutual inductance of the designed structure is simplified as follows:

13 $$M_{improve} = M_{1} + M_{2} .$$

**Figure 24:**
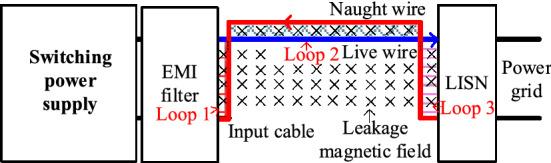
Schematic diagram of the negative coupling structure.

*M*_1_ and *M*_2_ are the mutual inductance between the adapter and rectangular plug Loop 1, Loop 2, respectively. Hence, the structure of the input plug can be optimized by adjusting the size of rectangular plug loops. The first method is adjusting the structure of rectangular plug Loop 2 to achieve negative coupling between adapter and Loop 2. Therefore *M*_*1*_ and *M*_2_ cancel each other out, as shown in Fig. 24. The second method minimizes the size of loop 1 and loop 2, so that *M*_*1*_ and *M*_*2*_ approaches 0.

**Figure 25:**
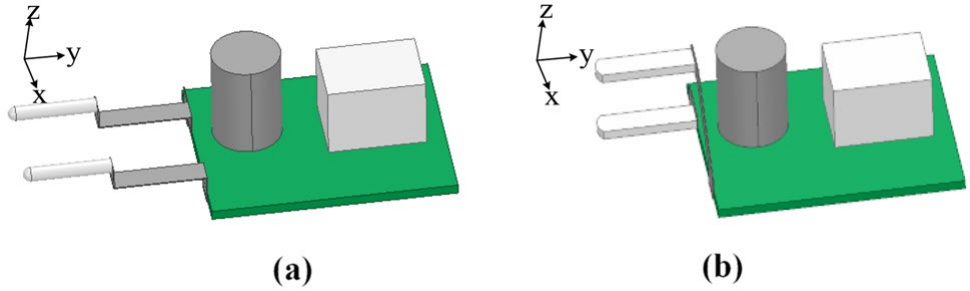
3D schematic diagram: (**a**) original structure; (**b**) proposed structure.

**Figure 26:**
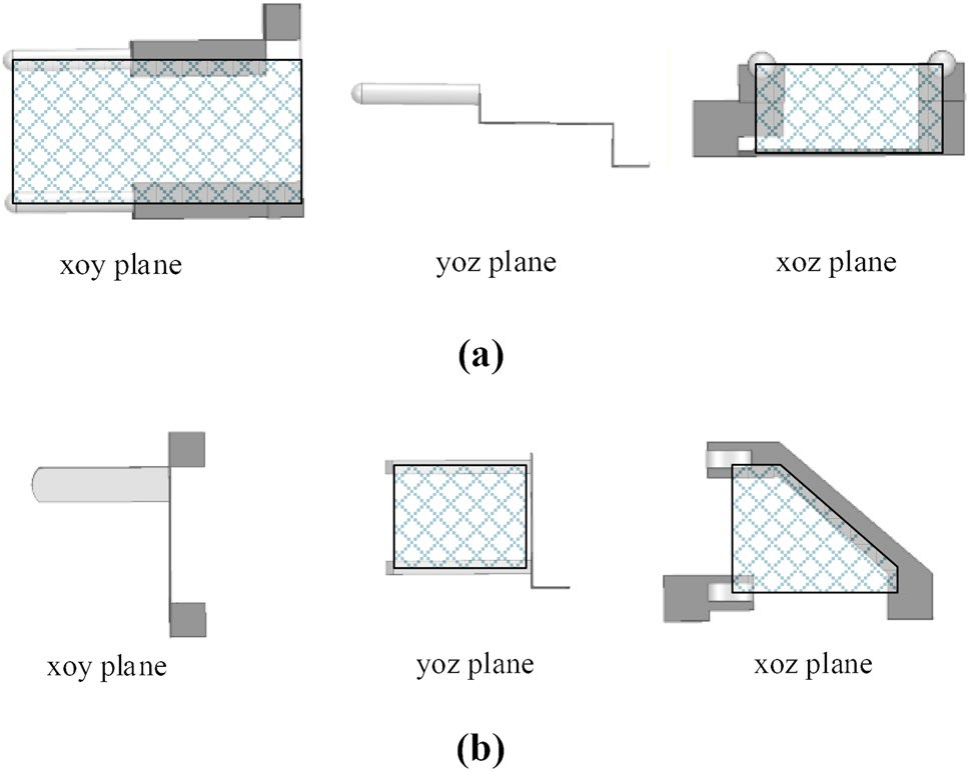
Three views of two structures in three planes: (**a**) original structure; (**b**) proposed structure.

In this paper, a structure with a minimum size of rectangular plug loops is designed to suppress the influence of near-field coupling. The prototype’s original and proposed structure is shown in Fig. 25, respectively. Moreover, the projections of the two structures on the xoy, yoz, and xoz planes are shown in Fig. 26. Compared with the original structure, more magnetic flux emitted by the adapter passes through the plug loop from directions perpendicular to the yoz plane and xoz plane, while the magnetic flux perpendicular to the xoy plane hardly passes through the plug loop. To minimize the effects of near-field coupling.

## Experimental validation

### The measurement result of mutual inductance

**Figure 27:**
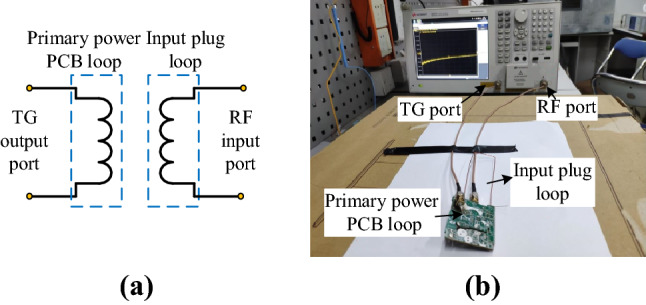
Mutual inductance measurement: (**a**) measurement approach of mutual inductance; (**b**) object diagram.

**Figure 28:**
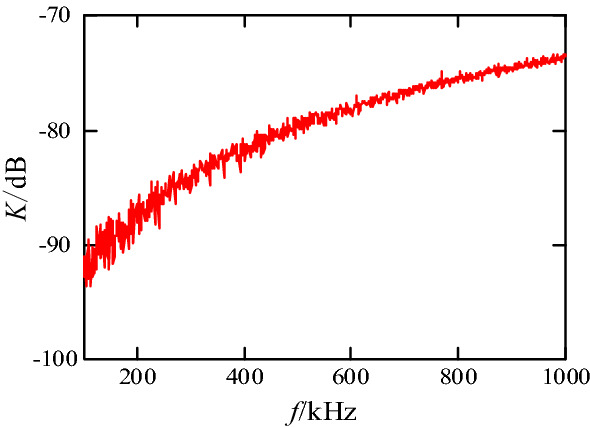
Insertion loss *K* curve between the primary power PCB loop and the plug loop.

The insertion loss method^16^ is used to measure mutual inductance between the primary power PCB loop and the input plug loop. After the MOSFET and bus capacitor *C*_*in*_ in the primary power PCB loop are short-circuited, the primary power PCB loop is connected to the TG output port of the network analyzer, and the input plug loop is connected to the RF input port, respectively, as shown in Fig. 27. Through circuit theoretical analysis and calculation, the relationship between the insertion loss (*K*) and mutual inductance is as follows:

14 $$\left| M \right| = \left| {\frac{{(50 + j\omega L_{pcb1} ) \cdot (50 + j\omega L_{ACin} )}}{100 \cdot j\omega }} \right| \times 10^{\frac{K}{20}} .$$

where *L*_*pcb1*_ and *L*_*ACin*_ are the self-inductances of the primary power PCB loop and the input plug loop, respectively. The insertion loss *K*(*f*) curve between the primary power PCB loop and plug loop is shown in Fig. 28. The same method is used to test the mutual inductance between the secondary power PCB loop and the input plug loop.

When the near-field coupling between the transformer and the input plug loop is evaluated, the secondary side of the transformer needs to be short-circuited. The mutual inductance between the transformer leakage inductance and the plug loop is calculated. The measurement results at *f* = 550 kHz are shown in Table 1.

**Table 1:** Measurement results of mutual inductance.

Item	Insertion loss *K*/dB	Mutual inductance *M*/pH
Primary power PCB loop	− 78.856	825.3
Secondary power PCB loop	Background noise	Very small and can be neglected
Transformer component of the primary winding	− 85.9	409.5

### Conducted EMI test results of prototype

**Figure 29:**
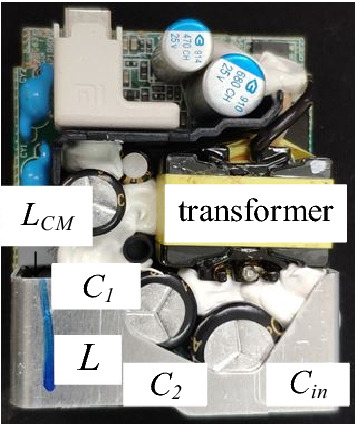
Front and component layout of the prototype.

A PD adapter is taken as the prototype for the experiment. The specification is shown in Table 2, and the physical drawing of the prototype is shown in Fig. 29.

**Table 2:** Main electrical parameters of the prototype.

Input voltage *V*_*in*_	Out voltage *V*_*out*_	Output power *P*_*out*_	Switching frequency *f*
AC220V/50HZ	DC24V	40 W	60 kHz

According to standard EMI measurement principles CISPR22, the prototype’s noise spectrums are tested in an electromagnetic shielding chamber. It is mainly composed of the device under test (DUT), ESH2-Z5 linear impedance stabilization network (LISN), and R&S ESCI EMI receiver.

**Figure 30:**
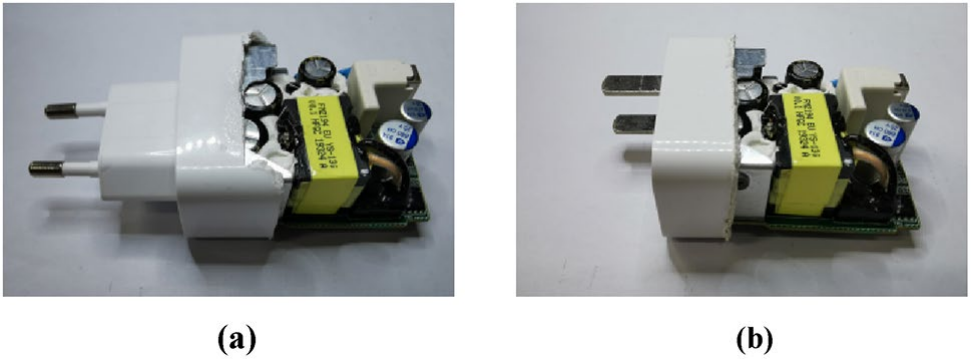
The realistic structure of the input plug: (**a**) original structure; (**b**) proposed structure.

The noise spectrums under the original and proposed structure are shown in Fig. 30.

**Figure 31:**
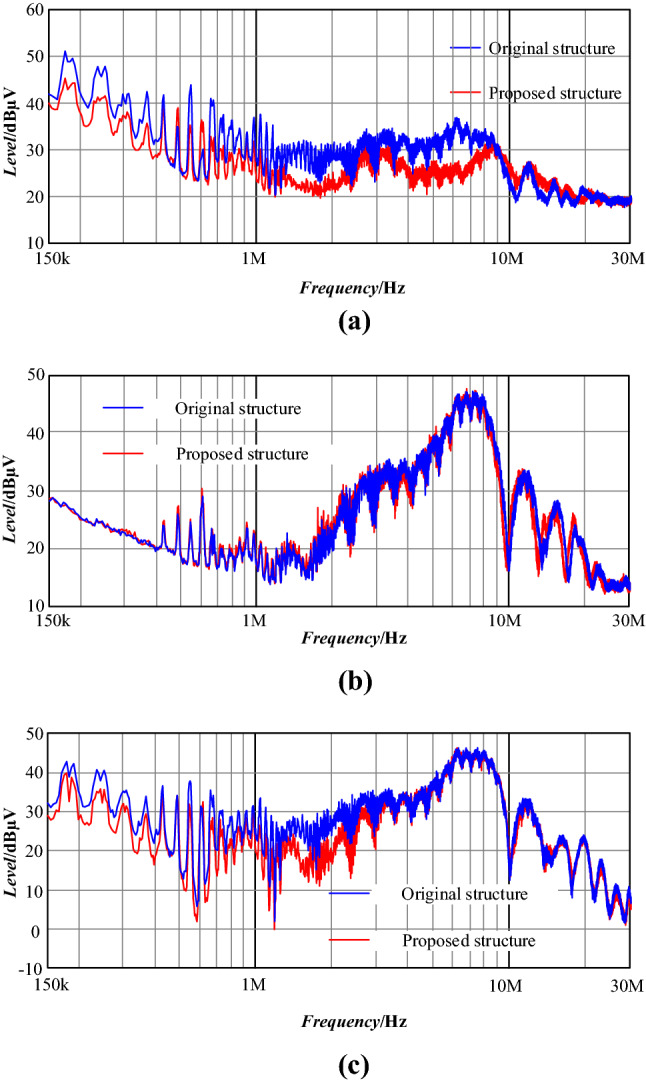
Noise comparison of the prototype: (**a**) DM noise; (**b**) CM noise; (**c**) total noise.

As shown in Fig. 31, compared with the original noise, DM noise is reduced by 8–10 dB from 150 kHz to 8 MHz, and CM noise is unchanged basically. The mutual inductance between the input plug loop and transformer as well as high-frequency power PCB loops is reduced by reducing the xoy plane projection area of the input plug, then near-field coupling is reduced, and the performance of DM conduction noise of the PD adapter in the low-frequency band is improved.

## Conclusion

In this paper, possible magnetic near-field coupling between components and loops in the PD adapter is analyzed, and the complete coupling model of the PD adapter is proposed. Then the mathematical model of the near-field coupling between plug loop and magnetic components as well as PCB traces is established. Based on it, a new structure is proposed which can effectively reduce the near-field coupling. Finally, a 40 W PD adapter is used as a prototype to verify the effectiveness and feasibility of the proposed optimization scheme.

## Data Availability

The authors declare that the data supporting the findings of this study are available within the article. All other relevant data are available from the corresponding author upon reasonable request.
